# Q&A: Where did the Neanderthals go?

**DOI:** 10.1186/s12915-017-0414-2

**Published:** 2017-09-01

**Authors:** Kelley Harris, Rasmus Nielsen

**Affiliations:** 1 Department of Genetics, Stanford University, Stanford, CA USA; 2 Department of Integrative Biology, University of California, Berkeley, California USA; 3 Museum of Natural History, University of Copenhagen, Copenhagen, Denmark

## Abstract

Genomic evidence has demonstrated that humans and Neanderthals interbred. Today, the genomes of most individuals outside Africa contain 2–3% Neanderthal DNA. However, it is still hotly debated why the Neanderthals went extinct and if humans contributed to the Neanderthal extinction. In this Q&A we explore what genomic data might have to say about this issue.

## In 2010 Svante Pääbo and colleagues published a paper showing that humans and Neanderthals interbred. Does that conclusion still hold true today?

Yes, more evidence of human/Neanderthal interbreeding has been building up every year. The initial 2010 finding by Pääbo and colleagues [1] was remarkable in that it was based on only a single ‘draft’ Neanderthal genome that was highly degraded but still contained useful genetic information. By 2014, however, Pääbo and colleagues had improved their ancient DNA sequencing techniques enough to produce a high coverage Neanderthal genome that is better quality than many modern human genome sequences [2]. By comparing modern human DNA to the high coverage Neanderthal genome and looking for regions of high similarity, computational biologists have identified thousands of chunks of modern human genomes that came from recent Neanderthal ancestors. Humans and Neanderthals have even been caught in the act of hybridization: when DNA was isolated from a 40,000-year-old human skeleton from Romania [3], that individual was shown to be a recent hybrid with a Neanderthal great-great-great-great-grandparent! The Neanderthal DNA in the modern human gene pool stems from interbreeding that occurred around 50,000 years ago, but there is also evidence that some gene flow from humans into Neanderthals occurred much earlier, closer to 100,000 years ago [4].

## But the Neanderthals are not here today. What do we know about when and how they went extinct?

From the fossil record, we know that the Neanderthal population started to decline around 40,000 years ago. There is some disagreement about how long they coexisted with humans—the species might have completely disappeared as long ago as 39,000 years ago, but some radiocarbon dating studies have suggested that Neanderthals might have survived in Asia as recently as 24,000 years ago. Many different factors probably contributed to their extinction, but their decline conspicuously coincides with the movement of anatomically modern humans into Europe and Asia [5].

## What theories have anthropologists then proposed that could explain the extinction?

Many different hypotheses have been proposed to explain the disappearance of Neanderthals. One possibility is competition with modern humans for resources or direct warfare between Neanderthals and humans. Humans were more technologically advanced than Neanderthals and might have been more efficient hunters. Anatomical evidence also suggests that humans were more efficient runners. It has also been proposed that Neanderthals were adapted to colder climates, potentially making it hard for them to thrive in the warming climate. Parasites and pathogens may likewise have contributed to the decline of Neanderthals. Like the conquistadors in the New World, humans may have carried diseases to which Neanderthals had built up no immunity.

## Does the genetic evidence have anything to say about possible causes of extinction?

There is strong genetic evidence that Neanderthal communities tended to be small and inbred by human standards, with little migration between populations that were situated in different parts of Europe. The Altai Neanderthal genome, which comes from Siberia, happens to be the genome of a girl whose parents were related at the level of half siblings. Such inbreeding may have left Neanderthals vulnerable to high rates of genetic diseases, which can threaten population health further and lead to death spirals of mutational meltdown. The structure of Neanderthal genetic variation tells us that the Altai girl’s inbred family history was not just a one-off event—rather, she came from a society that had maintained a small population size for tens of thousands of years. This is analogous to the way that human genomes contain evidence of an ‘out-of-Africa bottleneck’—African populations living today have more genetic diversity than non-African populations because the human population that first left Africa was relatively small and inbred. However, this migratory human population appears to have bounced back quickly. Neanderthals never rebounded like this; they just kept dwindling in population size until disappearing altogether from the fossil record.

## If Neanderthals were so genetically unhealthy, what did this mean for the children of the Neanderthals who interbred with humans?

**Fig. 1.**
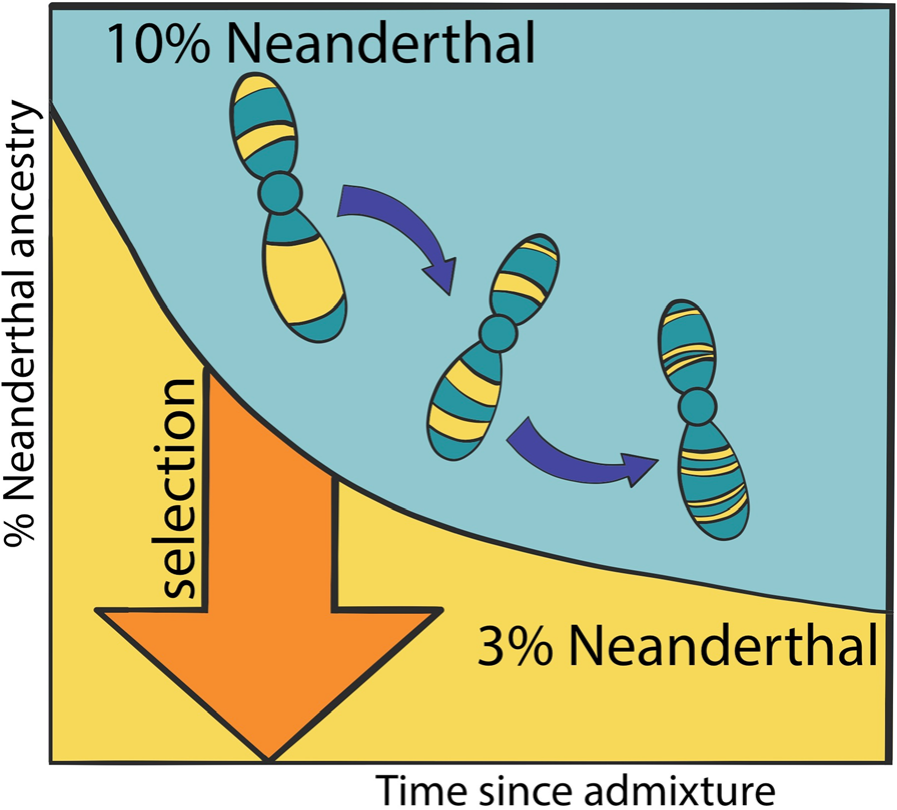
The decline of Neanderthal DNA in humans due to selection. The curve shows the expected decline in the proportion of Neanderthal DNA in modern humans due to natural selection based on the simulations in Harris and Nielsen [6]. The chromosomes depicted also illustrate the fact that not only is the Neanderthal DNA proportion decreasing through time, it is also distributed in smaller and smaller segments due to the effect of recombination

We recently found evidence that Neanderthal ancestry was probably a significant liability that hurt the fitness of early human–Neanderthal hybrids. At the time when Neanderthals first encountered anatomically modern humans, their long period of inbreeding made them at least 40% less fit than the modern humans who migrated into Eurasia to join them. Assuming that Neanderthals passed this fitness disadvantage down to their hybrid children, the fraction of Neanderthal ancestry in the human population is predicted to have decreased over time, as it was eliminated by natural selection. According to population genetic theory and simulations, Neanderthals may at one time have contributed around 10% of the human gene pool, though the contribution has been reduced to levels of only around 2–3% today [6, 7] (Fig. 1). Since humans outnumbered Neanderthals by approximately 10-to-1 during the interbreeding period, it is possible that Neanderthals did not truly die off at all, but simply melted together with the human species. One could perhaps argue that Neanderthals did not disappear due to warfare or competition—but due to love.

## This argument assumes that Neanderthal DNA has been removed from the human gene pool by natural selection—is there any evidence of that?

We can see evidence for selection against Neanderthal DNA by comparing the most important regions of the human genome to the ‘junk DNA’ that takes up space in our genome without helping to keep us alive. When Sriram Sankararaman, David Reich, Benjamin Vernot, and Joshua Akey used computational techniques to identify fragments of Neanderthal DNA in large modern human genome repositories, they found that these bits of Neanderthal DNA were much less likely to overlap important genes than we would expect to see if Neanderthal DNA was distributed at random [8, 9]. This suggests that people with Neanderthal DNA in certain important parts of their genomes have historically had fewer children on average than people whose Neanderthal DNA is harmlessly confined to ‘junky’ regions of the genome [10].

## Is there any empirical evidence that the proportion of Neanderthal DNA has decreased with time in humans?

A few days after we published our theoretical analysis of the dynamics of Neanderthal introgression, a team led by Qiaomei Fu and David Reich [11] published an empirical confirmation of one of our predictions! Specifically, they looked at Neanderthal ancestry in 51 anatomically modern humans who lived between 45,000 and 7000 years ago and discovered that the older individuals had more Neanderthal ancestry than the younger individuals did. Their study provides strong evidence that our ancestors had more Neanderthal ancestry than people have today, adding support to the idea that natural selection has been slowly purging Neanderthal DNA from our gene pool.

## How could this theory be tested further?

We need more estimates of the proportion of Neanderthal DNA in old human remains. The more of these data points we have, the better we will be able to estimate how much Neanderthal DNA was present in the human gene pool during the period of human–Neanderthal interbreeding, as well as the period immediately after this interbreeding stopped.

## Where can I found out more?

See references [5–8, 11].
